# Comparative transcriptome analysis reveals the genetic basis of coat color variation in Pashmina goat

**DOI:** 10.1038/s41598-019-42676-y

**Published:** 2019-04-23

**Authors:** Basharat Bhat, Ashutosh Singh, Zaffar Iqbal, Jai K. Kaushik, A. R. Rao, Syed Mudasir Ahmad, Hina Bhat, Aadil Ayaz, F. D. Sheikh, Shalini Kalra, Syed Shanaz, Masood Salim Mir, Pawan Kumar Agarwal, Trilochan Mohapatra, Nazir A. Ganai

**Affiliations:** 1 Department of Life Science, Shiv Nadar University, Gautam Buddha Nagar, UP 201314 India; 2 Division of Animal Genetics and Breeding, Sher-e-Kashmir University of Agricultural Sciences and Technology of Kashmir, Shuhama, Jammu and Kashmir 190016 India; 3 Animal Biotechnology Centre, National Dairy Research Institute, Karnal, India; 4 Division of Animal Biotechnology, Sher-e-Kashmir University of Agricultural Sciences and Technology of Kashmir, Shuhama, Jammu and Kashmir 190016 India; 5 Centre for Agricultural Bioinformatics, Indian Agricultural Statistics Research Institute, New Delhi, India; 6 Indian Council of Agricultural Research, New Delhi, India

**Keywords:** Bioinformatics, RNA sequencing, Genetic databases

## Abstract

The genetics of coat color variation remains a classic area. Earlier studies have focused on a limited number of genes involved in color determination; however, the complete set of trait determinants are still not well known. In this study, we used high-throughput sequencing technology to identify and characterize intricate interactions between genes that cause complex coat color variation in Changthangi Pashmina goats, producer of finest and costly commercial animal fiber. We systematically identified differentially expressed mRNAs and lncRNAs from black, brown and white Pashmina goat skin samples by using RNA-sequencing technique. A pairwise comparison of black, white and brown skin samples yielded 2479 significantly dysregulated genes (2422 mRNA and 57 lncRNAs). Differentially expressed genes were enriched in melanin biosynthesis, melanocyte differentiation, developmental pigmentation, melanosome transport activities GO terms. Our analysis suggested the potential role of lncRNAs on color coding mRNAs in *cis* and *trans* configuration. We have also developed online data repository as a component of the study to provide a central location for data access, visualization and interpretation accessible through http://pcd.skuastk.org/.

## Introduction

Coat color variation is regarded as the ensign of domestication^1^ and a defining characteristic of some breeds. Apart from the aesthetic appeal, coat color in domestic animals has many fold significance. Intriguingly, the genes responsible for the pigmentation are also pleiotropic in their effect on traits affecting animal health. Uncovering the genetics of coat color is of paramount importance for genomic selection and molecular breeding of fur and fiber-bearing animals. Interestingly, the abounding diversity in mammalian coat color has a deceptively simple origin linked to the type of melanin produced in melanocytes. There are two types of pigments eumelanin and pheomelanin^2^. The coat color variation results from a complex interplay of the produced melanins, their ratio (eumelanin and pheomelanin), the intensity of the pigmentation and the distribution of the pigment along hairs. The coat color of an animal is determined by the distribution and activity of melanocytes in the body.

Studies to decipher the genetics of coat color in mammals began right at the dawn of 20th century immediately following the rediscovery of Mendel’s Laws^3^. Most of the information comes from experiments on mice and rat and more than 150 genes affecting coat color have been discovered so far^1^. Three major mechanisms defining coat color variation have been identified; i) melanocyte migration and differentiation ii) melanocyte signaling and regulation and iii) melanin production and transport. Major genes have been discovered for spatial and temporal pigment-type switching (*MC1R, ASIP, DEFB300*), pigment diluting (*TYR, TYRP1, PMEL17*) and genes responsible for migration and survival of melanocytes (*BTA3, KIT, MITF, KITLG*)^3^. The current challenge to identify and characterize intricate interactions between genes to cause complex color variations needs a deeper insight. Recently the hunt for genetic networks responsible for the coat color has relied on RNA-sequencing in sheep^4^ and dog^5^. Whole transcriptome sequencing has enabled the discovery of all the coding and non-coding^6^ transcriptional units leading to understanding the global and temporal control of genetic networks and the ensuing processes.

For wool and fur type animals the coat color is one of the most defining economic traits. Understanding the gene networks and metabolic pathways controlling the coat color should help in devising effective breeding strategies for high quality and quantity of precious animal fiber like Pashmina. In the present study, we investigated the differentially expressed transcripts (mRNA and lncRNA), the relationship between lncRNAs with their target mRNA (in *cis* and *trans* configuration) and explored pathways and biological processes that might have caused the variation in the Pashmina coat color. The results provided several candidate genes and lncRNAs that might play an important role in the Pashmina coat color and pigmentation. Some of the highly differentially expressed genes (DEGs) were validated by qPCR.

## Materials and Methods

### Ethics statement

This study was approved by Animal Welfare and Ethics Committee of the Sher-e-Kashmir University of Agricultural Science and Technology of Kashmir (SKUAST-K). As per the guidelines of the committee, the skin samples were collected aseptically using biopsy punch under local anesthesia with minimal pain and discomfort to the animal.

### Experimental design and sampling

Six Pashmina goats (two brown, two white and two black) of the same age (26 months) and sex (doves) were selected for sampling from the SKUAST-Kashmir goat farm located in the northern Himalayas (Satakna, Ladakh) at an altitude of 12000 feet above mean sea level. The skin samples were collected from the flanking region of each goat at the same time point. Prior to sampling, the sampling site was sheared, shaved and locally anesthetized with 2% lignocaine. Approximately 7 mm diameter skin samples were harvested aseptically with a single-use skin biopsy punch. The skin samples were rinsed in the DEPC treated water, chopped with a fresh surgical scalpel blade and then transferred into the microfuge tubes containing RNA later solution. The samples were incubated with RNA-later solution for three hours at room temperature and then, transferred to the liquid nitrogen container till further processing.

### Total RNA extraction, library construction and sequencing

Trizol reagents (Invitrogen, USA) was used to extract total RNA from skin samples as per the manufacturer’s guidelines. RNA samples with RIN value greater than 8.0 and an OD 260/280 ratio greater than 1.7 were selected for RNA-sequencing. For subsequent library preparation and sequencing RNA samples were stored at −80 °C. Approximately 4 µg of total RNA was used to prepare the RNA sequencing library using the TruSeq RNA Sample Prep Kits (Illumina) as per the kit protocol. Agilent-tape station plots were used at every step to assess mRNA quality, enrichment success, fragmentation sizes, and final library sizes. Finally, the amplified fragments were sequenced using Illumina HiSeq^TM^ 2500 to obtain 2 × 100 bp paired-end reads.

### Mapping reads to the reference genome

The raw reads from all six samples were cleaned by removing the adapters, low-quality reads and those containing undermined bases. Cleaned reads from all samples were subjected to quality control analysis with FastQC^7^. The cleaned reads (quality scores >20 and length >25) were aligned to the reference genome of domestic goat^8^ (*Capra hircus*) downloaded from National Centre for Biotechnology Information – NCBI. The Bowtie program v2.2.6^9^ was used to build the index of goat reference genome, whereas the reads were assembled by using the Tophat program v2.1.0^10^. We allowed up to 1-mismatches in the seed region and reported all the multiple mapped positions. Of all the filtered reads, about 82–87% of reads in all the samples were properly aligned to the reference genome.

### Identification of differential expressed genes and gene enrichment analysis

Cufflinks package v2.2.1^11^ was used for transcripts assembly and abundance measurement. Each assembled transcript was expressed as fragments per kilobase of exon per million (FPKM) fragments mapped. Finally, we estimated relative gene expression and identified DEGs by using CuffDiff program. DEGs between three sets of samples were screened based on the threshold of P-value < 0.05 and absolute log2 (fold change) ≥1. Significantly, dysregulated genes were mapped to each term of GO database^12^. The hypergeometric test was used to identify significantly enriched GO terms against genome background (*Bos taurus*), where Bonferroni corrected P-value < 0.05 was used as a threshold. To further evaluate the biological significance of the DEGs, the KEGG pathway enrichment analysis was performed.

### Coding potential calculation and lncRNA-target gene prediction

To achieve high-quality data, we utilized *Coding-Non-Coding-Index* (CNCI)^13^, *Coding potential calculator* (CPC)^14^, *PFAM*^15^, and *PLEK*^16^ to distinguish long non-coding RNA from mRNA. These programs are based on different philosophies to distinguish coding and non-coding sequences. Transcripts anticipated with coding potential by any of the four tools were filtered out, and final dataset contained transcripts shared by four tools as potential lncRNAs.

To investigate the function of identified lncRNAs, we predicted their target genes in the *cis* and *trans* configuration using the in-house developed Perl scripts. We sought coding genes in the range of 100 kb upstream as well as downstream of the identified lncRNA for *cis* role. The *trans* role alludes the impact of lncRNA on mRNA at the expression level.

### Confirmation of differentially expressed mRNAs with qPCR

To confirm the RNA-sequencing analysis results, we selected nine genes which are primarily related to melanin biosynthesis and pigmentation for validation through quantitative reverse transcription PCR (qPCR); these genes included *ASIP, GNAQ, WNT3A, KIT, KITLG, PMEL, TRY, TRYP1* and *DCT*. cDNA was synthesized from 0.5 μg of the same total RNA used in RNA sequencing using the Revert Aid First Strand cDNA Synthesis Kit (Thermo Scientific, USA) as per the manufacturer’s protocol. qPCR reactions were run on Roche Lightcycler 480 II in a 20-μl reaction containing 0.5 μl of cDNA template, 10 μl of 2× SYBR Green Master Mix, 0.3 μl of each primer (10 μmol/μl) and nuclease free water 8.9 μl. The amplification program consisted of one cycle at 95 °C for 10 s, followed by 40 cycles of 95 °C for 15 s and 55 °C for 34 s. The qPCR reactions for each gene were performed with three replicates. Relative gene expression was normalized to the expression of goat *GAPDH* and fold change were calculated with the 2^−ΔΔCT^ method^17^. *GAPDH* were initially tested and shown to be stable under the experimental conditions. The primers used for the qPCR are listed in Supplementary Table 1.

### Pashmina coat color database

All data are made available online through an intuitive, user-friendly and interactive web interface running on XAMPP server. PHP v5.6 and Perl v5.6 were used for server-side programming, MySQL v5.7 for data storage and AJAX (Asynchronous JavaScript and XML) for data retrieving from the database.

## Results

### Alignment of sequencing reads

From six samples, we obtained a total of 60.20 Gb RNA-seq data, all sequence data were at 2 × 100 bp length (43,868,700 to 59,356,541 PE reads per sample). Cleaned reads were aligned to the *Capra hircus* genome assembly downloaded from NCBI server by using the TopHat2 programs. Quality control and alignment statistics provided in Supplementary Table 2 suggest sequencing data were of high quality and uniform among all sets of samples. The percentage of reads mapped to the reference genome were similar between all groups suggesting that there were no sequencing biases in the data.

### Differentially expressed coding and long non-coding RNA

**Figure 1:**
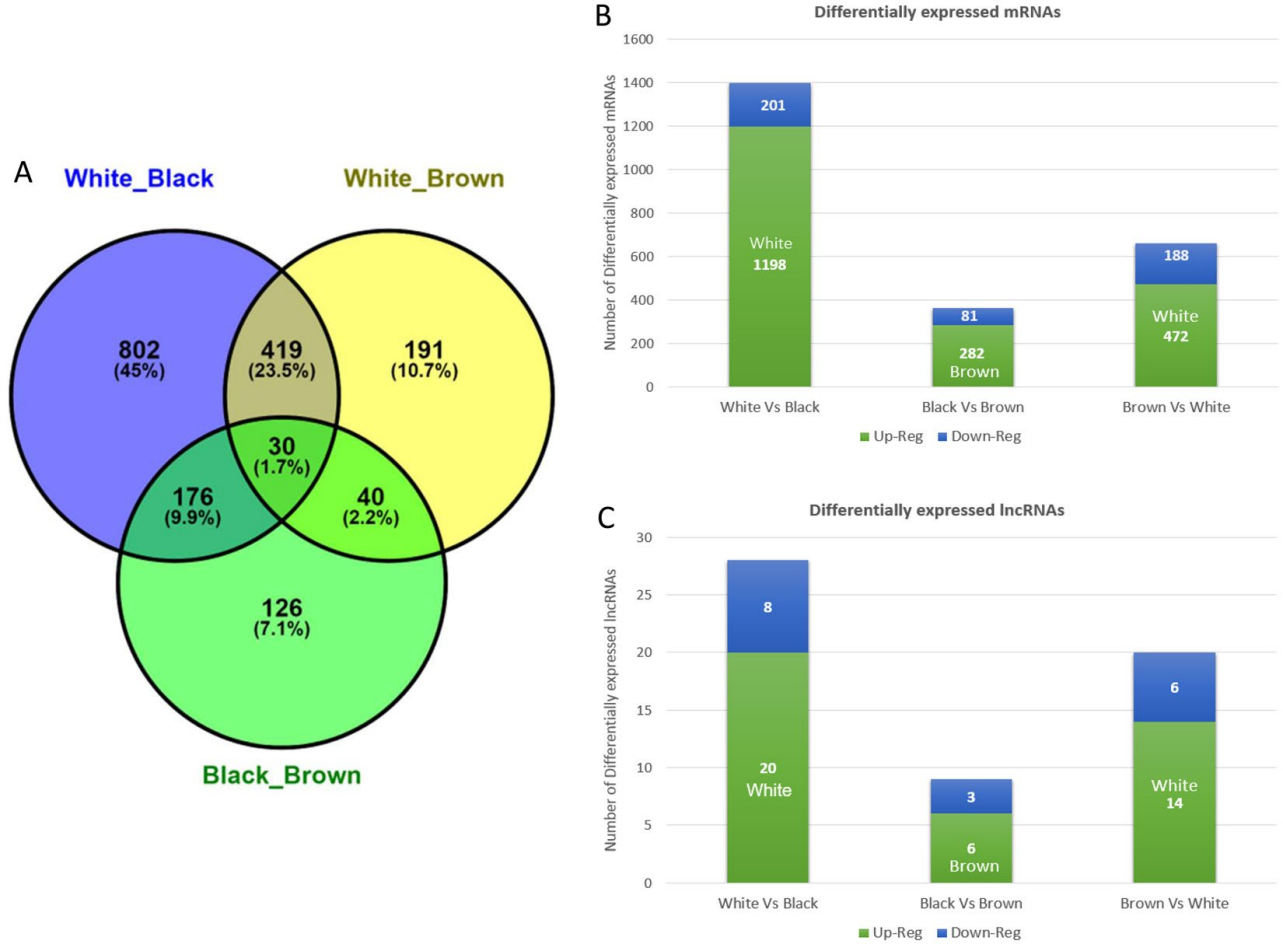
RNA-seq analyses of DEGs in different coat colour of Pashmina fiber. (**A**) Venn diagram representing DEGs between different groups. (**B**) Showing the overlap of differentially expressed mRNA between groups. (**C**) Showing the overlap of differentially expressed lncRNA between groups.

We obtained a total of 2479 DEGs, of which there were 57 lncRNA and 2422 mRNA (Supplementary Table 3) from the pairwise comparison of samples (i.e. black vs white, white vs brown and brown vs black) as shown in Fig. 1. In addition, approx. 7% (171 transcripts) of mRNAs were unclassified and unannotated (Supplementary Table 3). Supplementary Table 4 summarizes list of genes that may mediate fiber coloring in Pashmina goats.

### Functional enrichment analysis of mRNA in Pashmina coat color

Supplementary Tableslore the biological function and investigate the over-representation of DEGs in a given gene list, we performed GO enrichment analysis. The 372 transcripts in the list of black vs brown coat color, 680 transcripts in the brown vs white coat color and 1427 in the White vs black coat color that were differentially expressed in the respective pairs were mapped to the Gene Ontology database. A total of 365 GO terms were obtained by using the FDR corrected P-Value < 0.05 (Supplementary Table 5). DEGs were classified into three main categories: biological process (BP), cellular components (CC) and molecular function (MF). Important GO terms have been listed in Table 1. This analysis indicated that the identified DEGs are mainly involved in phenolic compound biosynthetic processes (GO.0046189), single-organism cellular processes (GO.0044763), regulation of biological processes (GO.0050786), melanin biosynthesis processes (GO.0042438), regulation of multicellular organismal processes (GO.0051239), inflammatory responses (GO.0006954) and pigment biosynthesis processes (GO.0046148).

**Table 1:** Gene Ontology (GO) terms for differentially expressed genes.

Category	GO ID	GO Term	Contrast Group					
			White V/s Black		White V/s Brown		Brown V/s Black	
			*Count*	*P-Value	*Count*	*P-Value	*Count*	*P-Value
BP	GO.0046189	*phenol-containing compound biosynthetic process*	8	0.0	8	0.0	—	—
BP	GO.0042438	*melanin biosynthetic process*	6	0.000563	4	0.0011	—	—
BP	GO:0048066	*developmental pigmentation*	6	0.016	—	—	4	0.015
BP	GO:0006582	*melanin metabolic process*	6	0.00108	4	0.0017	2	0.08
BP	GO:0043473	*pigmentation*	9	0.00551	—	—	5	0.013
BP	GO.0046148	*pigment biosynthetic process*	7	0.011	4	0.024	—	—
BP	GO:0030318	melanocyte differentiation	4	0.0405	—	—	4	0.006
BP	GO.0002376	*immune system process*	82	0.0	57	0.0	22	0.027
BP	GO:0060070	canonical Wnt signaling pathway	16	0.0016	—	—	—	—
BP	GO.0044763	*single-organism cellular process*	359	0.0	182	0.0	80	0.049
BP	GO.0044700	*single organism signaling*	192	0.0	83	0.0	46	0.034
BP	GO.0007154	*cell communication*	195	0.0	85	0.0	46	0.04
BP	GO.0042127	*regulation of cell proliferation*	177	0.0	39	0.0	17	0.022
BP	GO.0007166	*cell surface receptor signaling pathway*	102	0.0	48	0.0	26	0.009
BP	GO.0050789	*regulation of biological process*	183	0.0	90	0.0	40	0.034
BP	GO.0065007	*biological regulation*	345	0.0	163	0.0	40	0.0341
BP	GO.0014033	*neural crest cell differentiation*	8	0.0072	—	—	4	0.036
BP	GO.0006954	*inflammatory response*	31	0.0	21	0.0	11	0.010
CC	GO.0033162	*melanosome membrane*	3	0.03741	3	0.0043	—	—
CC	GO.0044425	*membrane part*	213	0.0	111	0.0	—	—
CC	GO.0044464	*cell part*	420	0.0	76	0.0	33	0.034
CC	GO.0045009	*chitosome*	3	0.0374	3	0.0043	—	—
CC	GO.0005576	*extracellular region*	159	0.0	101	0.0	38	0.025
CC	GO.0031224	*intrinsic component of membrane*	188	0.0	102	0.0	—	—
CC	GO:0042470	melanosome	8	0.04761	5	0.03	—	—
CC	GO:0048770	*pigment granule*	8	0.0476	5	0.033		
MF	GO:0004896	cytokine receptor activity	8	0.0121	—	—	—	—
MF	GO:0005044	*scavenger receptor activity*	6	0.0191	5	0.0024	—	—
MF	GO:0098772	*molecular function regulator*	45	0.0039	—	—	—	—
MF	GO:0044877	*macromolecular complex binding*	38	0.036	—	—	—	—
MF	GO:0060089	*molecular transducer activity*	16	0.0061	31	0.0016	—	—
MF	GO:0005488	*binding*	360	0.0	169	0.0	84	0.0499
MF	GO:0005515	protein binding	186	0.0	84	0.0	—	—
MF	GO:0043167	ion binding	139	0.0	22	0.0	40	0.0065
MF	GO:0046872	metal ion binding	129	0.0	61	0.0	34	0.037

Note: ‘BP’, ‘CC’ and ‘MF’ represent biological process, cellular component and molecular function, respectively.

*P-Value is FDR corrected P-value.

#### KEGG pathway enrichment analysis

**Figure 2:**
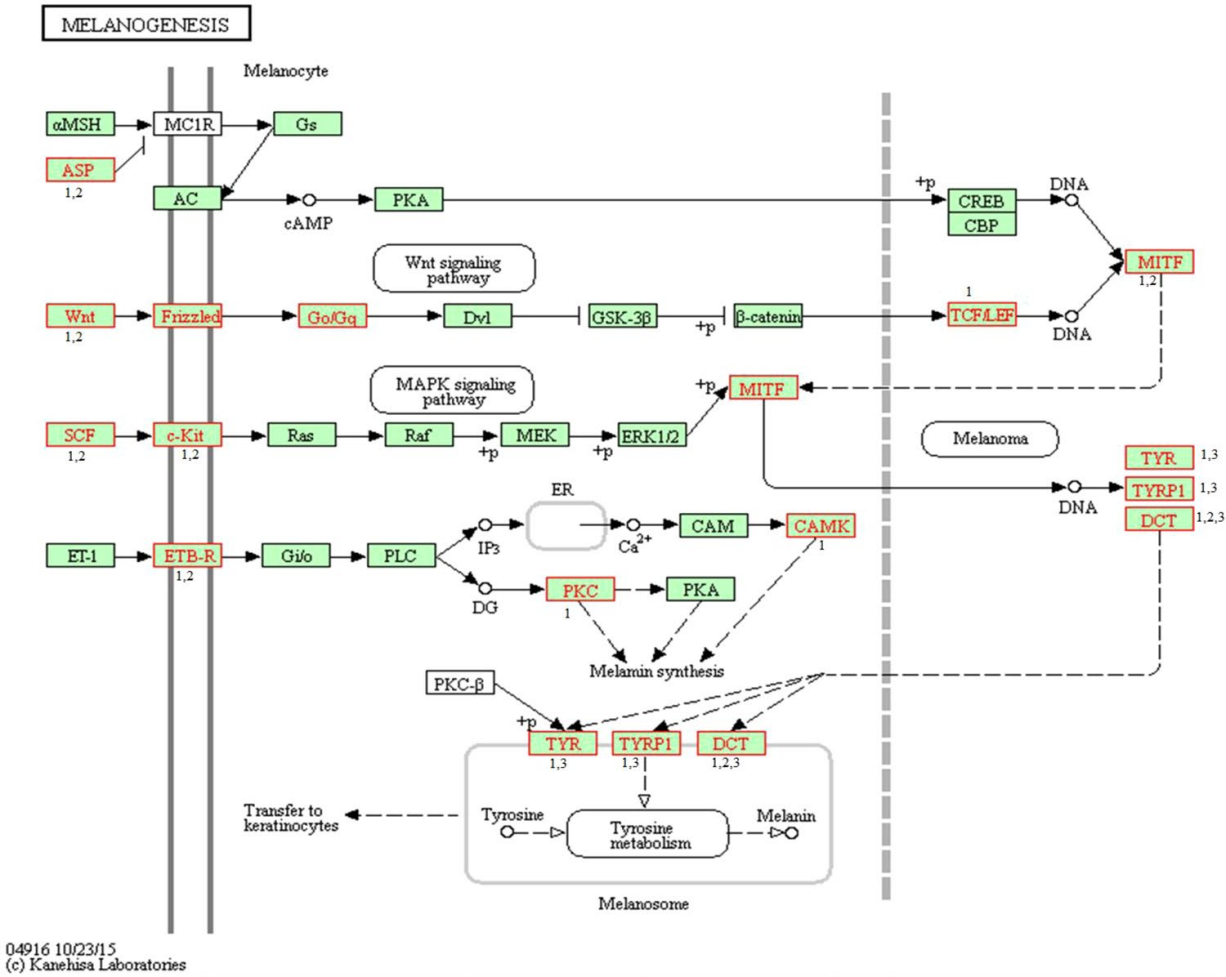
Differentially expressed coat color genes in Changthangi Pashmina goat and their involvement in melanogenesis signaling pathway (Adapted from ref.^18^). DEGs are shown in red coloured frames. Number (1, 2, and 3) represents contrast groups. 1 Black Vs White. 2 Black Vs Brown. 3 White Vs Brown.

**Figure 3:**
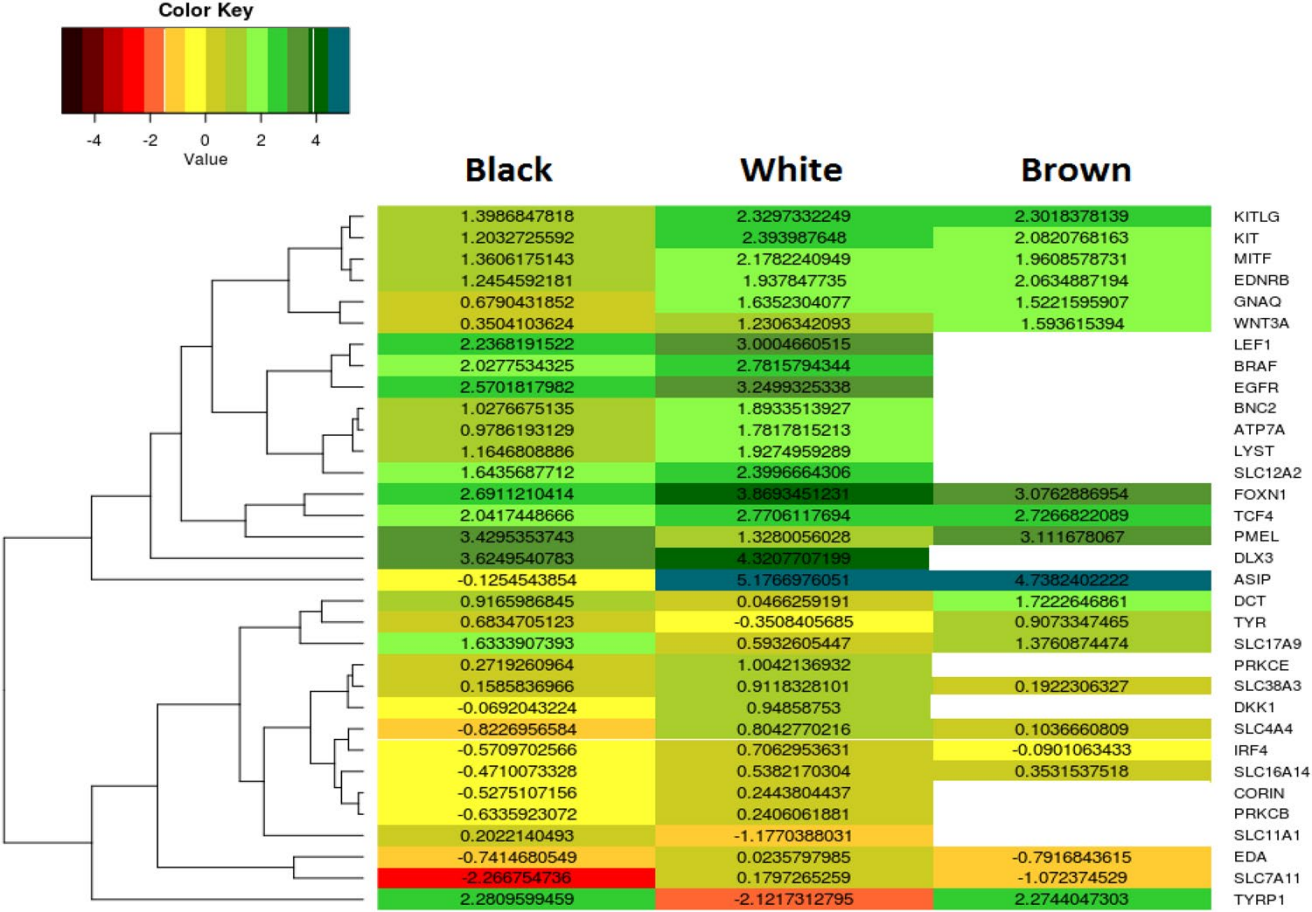
2D – Hierarchical clustering analysis exploring the difference in gene expression between White, Black and Brown Pashmina fiber. Each row in the map represents a differentially expressed gene and column represents condition used. Log10(FPKM) value is used for constructing heat-map. Blank space specifies gene is not significantly expressed in a particular sample.

Based on a FDR corrected p-Value < 0.05, KEGG enrichment analysis revealed that the DEGs were involved in a total of 20 pathways (Supplementary Table 5), which included signaling pathways like Chemokine signaling pathway, MAPK, Hippo, mTOR, RAP1, RAS and Wnt. Other important pathways associated with the coat color regulation and pigmentation included cytokine-cytokine receptor interaction, tyrosine metabolic pathway, melanogenesis, and melanoma pathways. In this study, we emphasized on melanogenesis pathway. A total of 33 DEGs were involved in melanogenesis pathway chx:04916 Fig. 2^18^, which may have a potential role in the Pashmina fiber coloring. Figure 3 shows the two-dimensional hierarchical clustering of 33 DEGs involved in melanogenesis pathway.

### Validation of RNA-seq results with qPCR

Among all DEGs, we selected nine genes specifically *ASIP, GNAQ, WNT3A, KIT, KITLG, PMEL, TRY, TRYP1* and *DCT* which are related to melanin synthesis to validate the expression levels through qPCR. The expression levels of genes by qPCR and RNA-seq were highly correlated (Pearson’s correlation coefficient = 0.87) thus validating the RNA-seq result (Supplementary Fig. 1).

### Genomic features of lncRNAs

**Figure 4:**
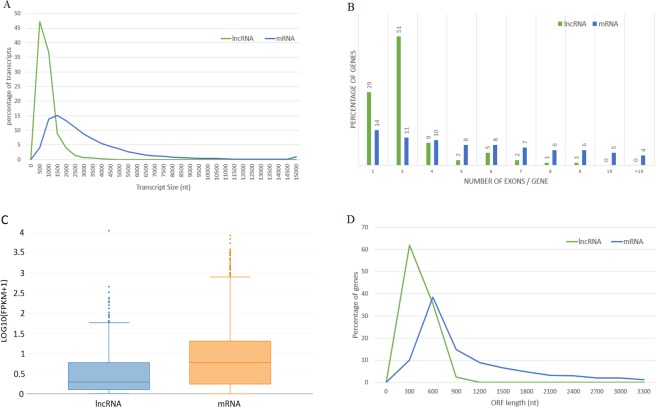
Comparison of expression and genomic features of predicted lncRNA and mRNA in Pashmina goat. The lncRNA and mRNA identified in this study are utilized to identify few primary differences between two classes. (**A**) Distribution of transcript length of long non-coding and coding transcripts in Pashmina goat skin. The X-axis/horizontal axis represents the length of transcripts and Y-axis/vertical axis represents percentage of genes. LncRNAs were shorter in length than mRNAs with 0.7 kb and 3 kb average length of each transcript, respectively. (**B**) Distribution of number of exons present in mRNA and lncRNA, most of lncRNA (80%) have 2 or 3 exons whereas mRNAs tend to have two or more exons. Due to the limitation of algorithms single-exon lncRNAs were filtered out from the goat genome. (**C**) Expression levels indicated by LOG10(FPKM + 1) in the mRNA and lncRNAs. (**D**) Represents distribution of ORF in mRNA and lncRNAs. LncRNAs have shorter ORF in length than mRNAs with a mean of 100 bp and 400 bp, for lncRNAs and mRNAs, respectively.

In the present study, we have identified a total of 665 multiple-exon lncRNAs from the brown, black and the white skin samples among which 57 lncRNA were significantly dysregulated. To exhaustively inspect the differences between the lncRNAs and mRNAs, comparative analysis of gene structure and expression were performed between 665 lncRNAs and 30956 mRNAs from all samples. Our results showed that 1) there was a distinct divergence in the distribution of transcript length between mRNA and lncRNA that is consistent to the previous studies^19^ (Fig. 4A), 2) majority of lncRNAs contained less than three exons that was not the case for mRNAs (Fig. 4B), 3) lncRNA shows lower expression compared to that of mRNAs (Fig. 4C), 4) 70% of lncRNA contained shorter ORF, in contrast to that of mRNAs (Fig. 4D). Overall, our results showed that the predicted long non-coding transcripts containing two or three exons were shorter in length than the coding transcripts, which was also reported in other studies^19^. Open reading frames in lncRNAs were shorter in length (average size of 100 bp) than mRNAs (average size of 400 bp) that is consistent with previous observations^20^.

### Differentially expressed lncRNAs related to color coding mRNAs

Several long non–coding transcripts are known that act in *cis*–configuration by regulating the expression of the neighbouring genes usually in 100 kb upstream or downstream regions. To find the relationship between differentially expressed lncRNAs and their neighboring mRNAs, we searched protein-coding genes in 100 kb upstream and downstream regions of the DE lncRNAs. We identified a total of 227 protein-coding genes in the specified regions (Supplementary Table 6). Gene ontology enrichment analysis showed that lncRNA target genes in *cis* configuration were significantly enriched in 157 GO terms (Supplementary Table 6) which are mainly involved in cell differentiation (GO:0030154, corrected P-value = 7.60E-16), developmental process (GO:0032502, corrected P-value = 3.30E-11), peptide transport (GO:0015833, corrected P-value = 6.00E-11) and transcription from RNA polymerase II promoter (GO:0006366, corrected P-value = 5.20E-08). These findings supported the potential role of lncRNA in *cis*-configuration to regulate gene expression in cellular and dermal development.

LncRNA also work in *trans*-configuration and can control mRNAs that are far away in position on the same or different chromosome^19^. A total of 1493 (Supplementary Table 6) interactions were identified between 2422 protein-coding genes and 57 lncRNAs based on the correlation coefficient of their expression (|Pearson correlation coefficient| ≥ 0.95). Interaction of lncRNA with the genes related to melanin biosynthesis and pigmentation are shown in Supplementary Table 7. Gene ontology enrichment analysis showed that lncRNA target genes in *trans* configuration were significantly enriched in 406 GO terms (Supplementary Table 6) which encompasses a variety of biological, molecular and cellular processes. Importantly, we observed protein targeting (GO:0006605, corrected P-value = 1.20E-11), cellular protein complex assembly (GO:0043623, corrected P-value = 4.70E-12), calcium ion binding (GO:0005509, corrected P-value = 4.40E-15), protein binding (GO:0005515, corrected P-value = 5.20E-68) and gene silencing (GO:0016458, corrected P-value = 0.0011) were significantly enriched. KEGG enrichment analysis showed that lncRNA target genes in *trans* configuration were involved mainly in melanogenesis pathway (P-value = 2.76E-04), PI3K-Akt signaling pathway (P-value = 5.67E-06), cytokine-cytokine receptor interaction (P-value = 2.9E-4), MAPK signaling pathway (P-value = 2.61E-04) and ECM-receptor interaction (P-value = 3.44E-06). These observations suggest that lncRNAs in *trans*-configuration should have a potential role in regulating gene-expression related to melanogenesis.

### Web interface

Pashmina coat database has been thoroughly tested on the commonly available web-browsers like Google Chrome, Safari, Mozilla Firefox and Internet Explorer. It supports text query where user can input the gene name in the search box. The search results present the information on level of gene expression in different coat colors, gene function, pathway(s), protein class, GO terms and protein-protein interaction network. The primary objective of the web repository is to provide important genetic information on color/pigmentation development in fiber and skin of Pashmina goat, which is the producer of the finest and costliest Pashmina/Cashmere fiber. Snapshots of the database are illustrated in Supplementary Fig. 2 accessible through http://pcd.skuastk.org/.

## Discussion

We report a transcriptomic analysis of coat color variation in Pashmina goats. Our data suggest that coat color in Pashmina goat is largely controlled by conserved pigmentation pathways. We further investigated the potential role of lncRNAs in pigmentation and identified regulatory RNAs in the *cis* and *trans* configuration. We present an expression database on coat color genetics in Pashmina goat to the research community.

A brief overview of the pigmentation pathway is presented here because it is fundamental to discussion henceforth. Mammalian melanocytes produce two types of pigments inside mealanosome, eumelanin (black) and pheomelanin (red). L-Tyrosine is converted to L-DOPA quinone via L-DOPA by *TYR*. L-DOPA quinone is the bisecting point; in the absence of cysteine/glutathione, it leads to eumelanin synthesis via reactions catalyzed by *TRP-1* and *TRP-2*. The presence of cysteine/glutathione commences the alternate pathway to synthesize pheomelanin. The type of pigment produced is controlled by *MC1R* signaling. *MC1R* responding to two ligands of signaling molecules, the *α-MSH* and *ASIP*. *α-MSH* as *MC1R* agonist leads to the formation of eumelanin by increasing intracellular cAMP levels. *MC1R* interaction with *ASIP* bring down the levels of cAMP, causing the cells to become inactive and produce pheomelanin. Higher level of signaling directs higher expression of *TYR, TRP1, TRP2, OCA2* and *PMEL*, leading to increased eumelanin synthesis. Lower levels of signaling induce higher expression of cysteine transporter *SLC7A11*, leading to increased pheomelanins synthesis. *MYO5A*, *RAB27A, MLPH, MATP, SLC24A5* are involved in the transport of melanocytes.

We compared the transcriptomes of skin biopsies from three principal coat color variants found in Pashmina goats - black, white and brown. White coat transcriptome is characterized by upregulation of *ASIP, KIT, KITLG*, and *MITF*. *ASIP* was highly expressed in white (black vs white FC = 6.167, P-value = 0.01) and brown (black vs brown FC = 5.511, P-value = 0.01) coat color as compared to black. Higher expression of *ASIP* blocks the production of eumelanin by inhibiting the MSH-initiation pathway^2^. A mutation in *ASIP* causes the black pigmentation in pigs^21^; also, a missense mutation in *ASIP* gene is associated with the loss of white spotting pattern in donkeys^22^. Higher expression of *ASIP* gene in white and brown color further establishes its role as an antagonistic to *MC1R* gene. Intrestingly, *α-MSH* and *MC1R* do not show any difference in expression between different coat colors, which suggest *α-MSH* and *MC1R* were equally expressed  in all three Pashmina coat colors; however, *MC1R* and *ASIP* interaction inactivate melanocytes to produce pheomelanin. *KIT* gene codes for a transmembrane^23^ receptor involved in signal transduction by melanoblasts and *KITLG* (aka *MGF*) for its ligand. *KIT* gene is ascribed for white spotting and color sidedness^24^ and white color variation in cattle^25^. *KIT* gene is also a candidate gene for white spotting in domestic cats^26^. Mutation in the *KIT* gene is associated with variation in coat color in horses^27^. The higher expression of *KIT* and *KITLG* in white Pashmina goat skin transcriptome is consistent with the earlier studies and suggests that white color in Pashmina goat is linked to the *KIT* locus. *MITF* regulates the transcription of three major pigmentation enzymes: *TYR, DCT, TYRP1*. This gene is implicated in controlling white spotting in dogs^28^, degree of white spotting in Holstein cattle^29^ and white phenotypes in Geese^30^. In our study, upregulation of *MITF* in white skin suggest this gene may contribute to white phenotypes in Pashmina goats. White phenotype showed downregulation of enzymes for melanogenesis viz. *TYR, TRP1, TRP2* and those involved in the transportation of melanosomes, viz. *MYO5A, RAB27A, MLPH, MATP, SLC24A5* and reasonably so.

Color-coat transcriptome in black and brown goats is associated with the upregulation of enzymes involved in the mealanin production transport. The top upregulated genes are those coding for enzymes of melanogenesis, viz. *TYR, TRP1, TRP2*. *TYR* is a critical and rate-determining enzyme for the formation of melanin, tyrosinase-related protein 1 (*TRP1*) and DOPAchrome tautomerase (*DCT* or *TRP2*) are important enzymes that influence the quality and the quantity of melanin produced. Mutations or dysfunction of tyrosinase or its chaperone-like protein *TYRP1* leads to extensive hypopigmentation or Albinism^31^. White feather bulbs in ducks are ascribed mainly due to lack of *TYR* and *TYRP1* expression^32^. In our study, among the DE coat color genes, *TYR* and *TYRP1* showed the highest level of expression in the black compared to white skin samples. This is also consistent with pigmentation in domestic sheep^4^. *DCT* or *TYRP2* is involved in the isomerization of dopachrome to *DHICA*. It is plausible to think that the ethnic differences reflect differences in *DCT* expression^33^. Mutations of *DCT* dramatically decrease eumelanin content from melanocytes in mice^34^. In our study, *DCT* was significantly up-regulated in dark fiber color, with the highest expression in brown skin samples. Melanocyte protein or silver protein (*PMEL/SILV/PMEL17*) plays a central role in the structural organization of premelanosomes. PMEL acts as a scaffold in melanosomes by creating a proteolytic fibrillary matrix where melanin is deposited^35^. Mutation in SILV protein result in the dilution of pigmentation in many animals^36,37^. In our study, *PMEL* was significantly up-regulated in black and brown skin samples when compared to white skin (black vs white and brown vs white) as expected. The expression of all the genes involved in melanin production, trafficking and structural proteins for melanin has thus been validated in the current study on Pashmina goats.

Surprisingly in our study *WNT3A* and *FZD4* receptors that are the members of Wnt signaling pathway were found upregulated in the white skin and brown skin samples when compared to black skin. Wnt pathway is well documented for regulating hair follicle cycle. The canonical Wnt pathway has been reported for its important role in skin pigmentation and melanogenesis in chickens^38^. However, ascribing *WNT3A* role in mammalian coat pigmentation, we can’t rule out that such expression could also be because of the cyclicity of the follicles. Further studies are needed to validate any role of *WNT3A* in mammalian coat pigmentation.

In our study, we anticipated the potential function of lncRNAs in goat skin and identified regulatory mRNAs in *cis* and *trans* configuration. A cluster of nine closely related lncRNA target genes related to melanin production and melanin dilution (*TYR, TYRP1, PMEL*) have been identified (Supplementary Table 7). Also, three similar lncRNAs targets *DCT, TYRP1*, and *TYR* that are evolved from a common ancestral Tyrosinase gene were also identified, which suggests that the Tyrosinase family genes could be involved in the functional evolution of these lncRNAs. Furthermore, *FZD4* and *WNT3A*, which are the member of WNT signaling pathway, are regulated by ‘*LOC102186545*’ lncRNA in *trans* configuration, suggesting that lncRNAs are functionally conserved with respect to their targets.

We have developed an expression database on coat color genetics in Pashmina goat which provides researcher-friendly web interfaces to query, browse and visualize expression data across different coat colors. It also offers informative and flexible way to display gene expression profiles by specifying a gene name (e.g., *PMEL, TYR, ASIP*). To facilitate visualization of gene expression profiles, enriched GO terms and interactive heat-maps of pathways, scatter-plots, and chord-graphs are used. It is a unique portal for understanding essential biological processes and mechanisms underlying the complex agronomic traits in Pashmina goat. This web portal would provide information to researchers in devising an effective selection and breeding strategy for obtaining desirable coat color(s) that have better acceptability and fetch more prices for the ultra-fine Pashmina fiber.

## Conclusion

In this study, we generated mRNA and lncRNA expression profiles for white, black and brown Pashmina skin by using RNA-sequencing technology. This is perhaps the first report of mRNAs in conjunction with lncRNAs regulating coat color formation in Pashmina fiber; and thus, providing an opportunity for the molecular interventions for controlling the color trait in highly prized fiber like Pashmina.

## Supplementary information

Supplementary Tables and Figures

Supplementary Table 3

Supplementary Table 5

Supplementary Table 6

## Data Availability

The sequencing data were submitted to the Genome Expression Omnibus (GEO) database (Accession Number GSE107249) in NCBI.
