# Supplementary material for: Comparative transcriptome analysis reveals the genetic basis of coat color variation in Pashmina goat
Source: Sci Rep. 2019 Apr 23;9:6361. doi: 10.1038/s41598-019-42676-y (PMC6478727; doi:10.1038/s41598-019-42676-y)
Supplement: Supplementary file 1 — Supplementary Tables and Figures [file 41598_2019_42676_MOESM1_ESM.pdf]

Comparative transcriptome analysis reveals the genetic basis of coat color variation in Pashmina goat

Basharat Bhat[1], Ashutosh Singh[1], Zaffar Iqbal[2], Jai K Kaushik[3], AR Rao[5], Syed Mudasir Ahmad[4], Hina Bhat[4], Aadil Ayaz[2], F D Sheikh[2], Shalini Kalra[3], Syed Shanaz[2], Masood Salim Mir[2], Pawan Kumar Agarwal[6], Trilochan Mohapatra[6] and Nazir A. Ganai[2],*

[1]Department of Life Science, Shiv Nadar University, Gautam Buddha Nagar, UP, 201314, India
[2]Division of Animal Genetics and Breeding, Sher-e-Kashmir University of Agricultural Sciences and Technology of Kashmir,
Shuhama, Jammu and Kashmir, 190016, India
[3]Animal Biotechnology Centre, National Dairy Research Institute, Karnal, India
[4]Division of Animal Biotechnology, Sher-e-Kashmir University of Agricultural Sciences and Technology of Kashmir,
Shuhama, Jammu and Kashmir, 190016, India
[5]Centre for Agricultural Bioinformatics , Indian Agricultural Statistics Research Institute, New Delhi, India
[6]Indian Council of Agricultural Research, New Delhi, India

[*]Corresponding author
PhD Nazir A. Ganai
Division of Animal Genetics and Breeding, Faculty of F.V.Sc and A.H. Sher-e-Kashmir University of Agricultural Sciences and Technology of Kashmir Shuhama, Srinagar, India.
naganai@skuastkashmir.ac.in

Table 1. List of primers used in qRT-PCR validation

Name of the gene	Primer Sequence	Size
**ASIP**	F 5' CTGTCTCTATTGTGGCACTGAA 3'<br>R 5' AGCCTTTCTCTTGGAAGCTC 3'	89bp
**PMEL**	F 5' GATACCTTAGTCCTGGTGAAGC 3'<br>R 5' GGATCTCAGCACTCTCAATACC 3'	112bp
**KITLG**	F 5' CCCTTAGGAATGACAGCAGTAG 3'<br>R 5' AGCAAACCCGATCACAAGA 3'	119bp
**TYRP1**	F 5' CTACACTCCTCTCTCTGGGTTA 3'<br>R 5' CTCAATGGTAGCACACTCTCTC 3'	92bp
**KIT**	F 5' CCGTGGACTCTATGTGGATAAA 3'<br>R 5' GCCTTTCCTGACGGAGATAA 3'	105bp
**DCT**	F 5' GTTATGGCTGGAAAGGGATCT 3'<br>R 5' CACACGTCACACTCGTTACT 3'	99bp
**WNT3A**	F 5' GGCCACGCTACACCTACTTC 3'<br>R 5' GGTTGGGCTCGCAGAAGTTT 3'	84bp
**GNAQ**	F 5' TCAACGACGAGATTGAGCGG 3'<br>R 5' ATCAGAGTAGCCCGACCCAT 3'	146bp
**TYR**	F 5' CATGGTTCCTTTCATCCCTCTC 3'<br>R 5' GTCCGGTTCTGAATCTTGTAGG 3'	100bp
**GAPDH**	F 5' GGCGTGAACCACGAGAAGTA 3'<br>R 5' GGCGTGGACAGTGGTCATAA 3'	141bp

Table 2: Quality control and alignment statistics of RNA-seq reads to Capra hircus genome

Sample	Total Reads	Cleaned reads	Total mapped	Multiple mapped	Uniquely mapped
**Black_1 – PG_21**	48,215,917	45,220,488	38,264,989 (84.6%)	2,971,843 (7.8%)	35,293,146 (76.8%)
**Black_2 – PG_22**	43,868,700	39,842,480	34,293,638 (86.1%)	2,875,260 (8.4%)	31,418,378 (77.7%)
**White_1 – PG_11**	47,830,768	42,544,652	34,886,493 (82.0%)	2,632,621 (7.5%)	32,253,872 (74.5%)
**White_2 – PG_23**	59,356,541	56,594,841	47,508,418 (83.9%)	3,847,153 (8.1%)	43,661,265 (75.8%)
**Brown_1 – PG_24**	52,733,707	48,070,787	41,457,816 (86.0%)	3,329,677 (8.0%)	38,128,139 (78.0%)
**Brown_2 – PG_16**	49,035,207	43,897,474	37,969,989 (86.2%)	2,961,820 (7.9%)	35,008,169 (85.4%)

Table 4. Top differentially expressed colour coding genes in chanthangi Pashmina goat.

Gene	Sample 1	Sample 2	FPKM Sample1	FPKM Sample2	Log2FC	P-Value	Annotation
ASIP	black	white	0.88209	177.097	7.64938	0.0149	agouti signaling protein
	black	brown	0.88209	114.233	7.01682	0.0149	
PMEL	white	brown	3.77351	22.4587	2.5733	0.00115	premelanosome protein
	black	white	30.8623	3.77351	-3.03187	0.0004	
KIT	black	white	3.331	10.9571	1.71784	0.00005	KIT proto-oncogene receptor tyrosine kinase
	black	brown	3.331	8.02111	1.26785	0.00035	
KITLG	black	white	4.04987	10.2752	1.34322	0.0002	KIT ligand
	black	brown	4.04987	9.99253	1.30297	0.0006	
DCT	white	brown	1.04773	5.59719	2.41744	0.00015	dopachrome tautomerase
	black	brown	2.50077	5.59719	1.16233	0.0088	
	black	white	2.50077	1.04773	-1.25511	0.01855	
MITF	black	white	3.8986	8.83061	1.17956	0.00055	melanogenesis associated transcription factor
	black	brown	3.8986	7.10542	0.86596	0.0093	
WNT3A	black	brown	1.41965	4.92151	1.79356	0.00425	Wnt family member 3
	black	white	1.41965	3.4234	1.26989	0.0366	
EDA	black	white	0.47641	1.02386	1.10374	0.03845	ectodysplasin A
	white	brown	1.02386	0.45308	-1.17618	0.03105	
EDNRB	black	brown	3.47453	7.87339	1.18017	0.002	endothelin receptor type B
	black	white	3.47453	6.94379	0.99890	0.00905	
FOXN1	black	white	14.7482	47.911	1.69982	0.00005	forkhead box N1
	white	brown	47.911	21.6778	-1.14414	0.0007	
GNAQ	black	white	1.97199	5.13064	1.37949	0.0006	Guanine nucleotide-binding protein G(q) subunit alpha
	black	brown	1.97199	4.58211	1.21636	0.0019	
IRF4	black	white	0.56497	2.02647	1.8427	0.00375	interferon regulatory factor 4
	white	brown	2.02647	0.91383	-1.14896	0.042	
LEF1	black	white	9.3635	20.0949	1.10171	0.0036	lymphoid enhancer binding factor 1

LYST	black	white	3.2049	6.87228	1.10051	0.00025	lysosomal trafficking regulator
NGF	white	brown	1.02872	2.64157	1.36054	0.0333	nerve growth factor
ATP7A	black	white	2.66078	5.94043	1.15872	0.0008	ATPase copper transporting alpha
BNC2	black	white	2.79454	6.64159	1.24892	0.00015	basonuclin 2
BRAF	black	white	7.597	16.1445	1.08754	0.00125	B-Raf proto-oncogene, serine/threonine kinase
CORIN	black	white	0.590072	1.27683	1.11361	0.027	corin, serine peptidase
DKK1	black	white	0.933136	2.58206	1.46837	0.01675	dickkopf WNT signaling pathway inhibitor 1
EGFR	black	white	13.0682	25.7886	0.980678	0.0019	epidermal growth factor receptor(EGFR)
SLC11A1	black	white	1.22411	0.30819	-1.98985	0.01245	Iron transporter
SLC12A2	black	white	5.1736	11.0195	1.09082	0.00045	Sodium/Potassium/Chloride Transporter
SLC16A14	black	brown	0.624373	1.42355	1.18901	0.02775	Monocarboxylic Acid transporter
	black	white	0.624373	1.71295	1.456	0.00765	
SLC17A9	black	white	5.12121	1.80988	-1.50059	0.0012	Vesicular Nucleotide Transporter
	white	brown	1.80988	3.95938	1.12938	0.01675	
SLC38A3	white	brown	2.48888	1.21195	-1.03816	0.0254	Na(+)-Coupled Neutral Amino Acid Transporter 3
	black	white	1.17185	2.48888	1.08671	0.01565	
SLC4A4	white	brown	2.23508	1.10923	-1.01077	0.01165	Sodium Bicarbonate Cotransporter
	black	brown	0.439246	1.10923	1.33645	0.0045	
	black	white	0.439246	2.23508	2.34722	0.00005	
SLC7A11	white	brown	1.19689	0.342195	-1.8064	0.0124	Cystine/Glutamate Transporter
	black	white	0.103648	1.19689	3.52953	0.0131	
TCF4	black	brown	7.70404	15.2821	0.988151	0.00235	Immunoglobulin Transcription Factor 2
	black	white	7.70404	15.9684	1.05153	0.0012	
TYR	black	white	1.98074	0.704096	-1.4922	0.01935	Tyrosinase enzyme
	white	brown	0.704096	2.47771	1.81517	0.00545	
TYRP1	black	white	9.78607	0.119824	-6.35175	0.02575	Tyrosinase Related Protein 1
	white	brown	0.119824	9.72213	6.34229	0.0255	
PRKCB	black	white	0.530682	1.27202	1.26121	0.0175	Protein Kinase C Beta
	black	white	1.31249	2.72976	1.05646	0.0068	

CALR	black	white	629.946	347.842	-0.856797	0.03805	Endoplasmic Reticulum Resident Protein 60
	white	brown	347.842	605.862	0.800558	0.0484	
SEPT	black	white	9.0687	18.3496	1.01678	0.00125	Septin-11
	black	brown	9.0687	14.7001	0.696859	0.02325	
STIM1	black	white	14.0666	24.7633	0.815926	0.0078	Stromal Interaction Molecule 1
	black	white	2.57118	5.73746	1.15798	0.00315	
STIM2	black	brown	2.57118	5.16503	1.00635	0.00995	Stromal Interaction Molecule 2
TRPC1	black	white	1.03943	2.07962	1.00053	0.0451	Transient Receptor Potential Cation Channel Subfamily C Member 1

Fig. 1. Relative gene expression of selected differentially expressed genes (a – i).

Fold expression in qPCR

Fold expression in RNA-Sequencing

a

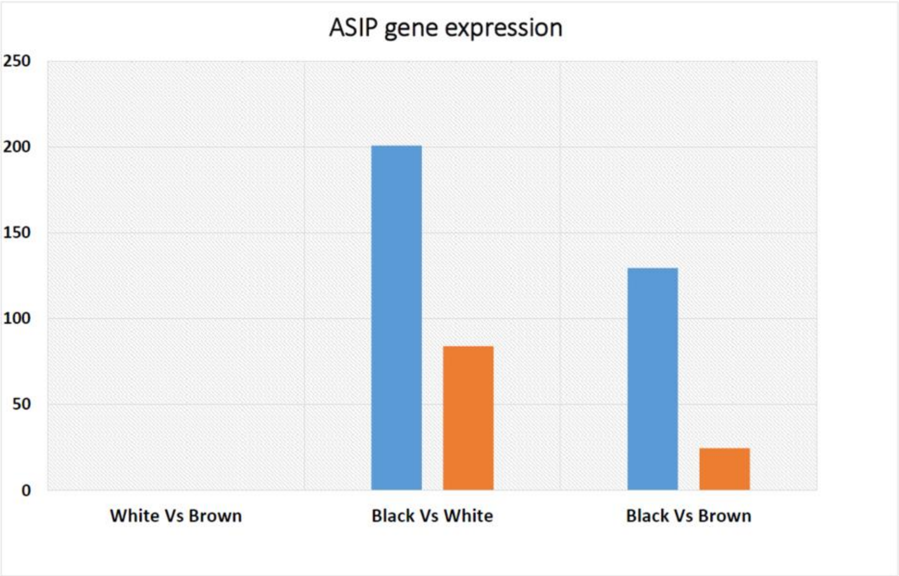

b

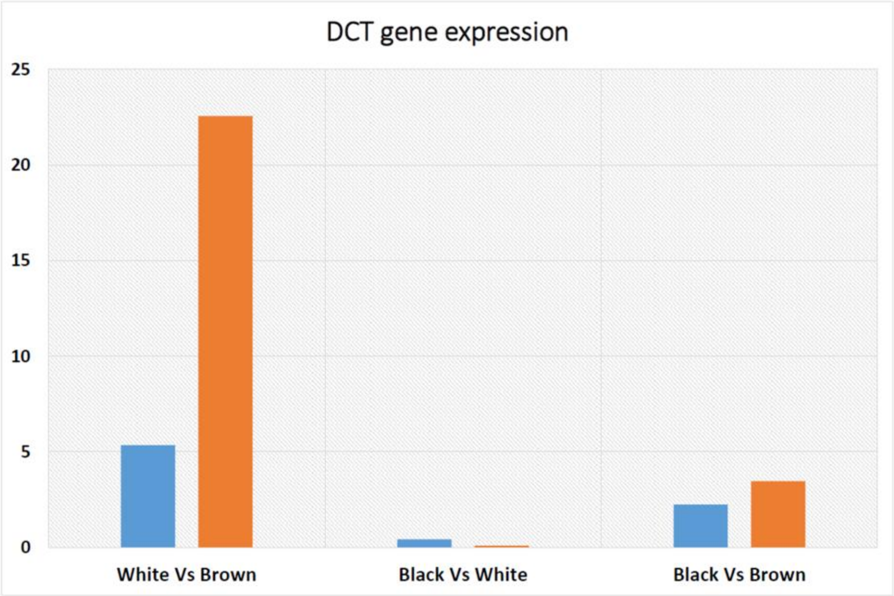

c

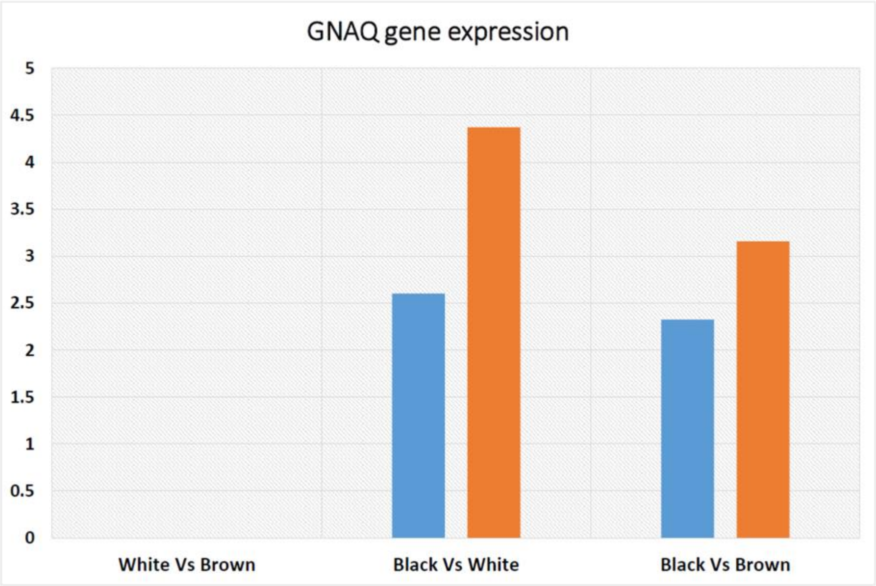

d

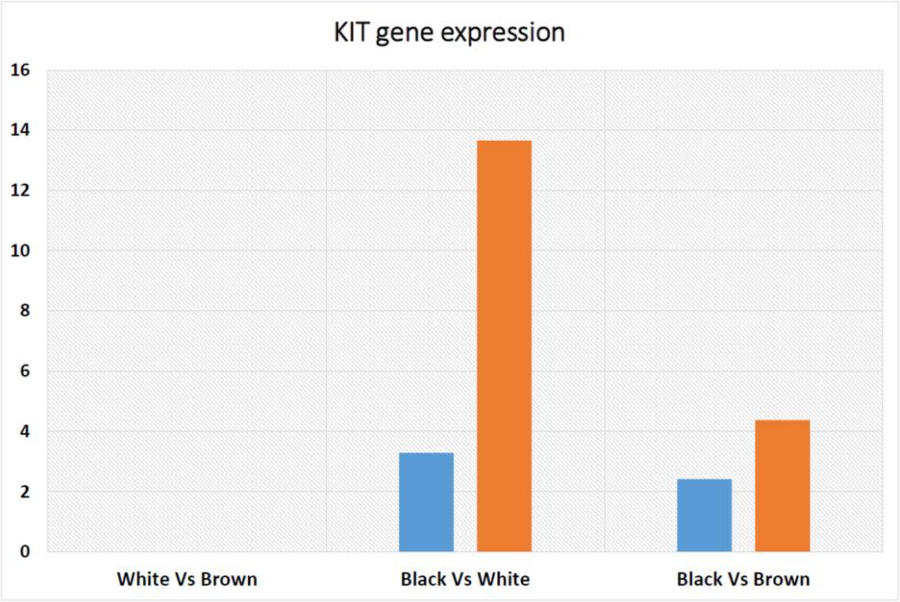
KIT gene expression
16
14
12
10
8
6
4
2
0
White Vs Brown
Black Vs White
Black Vs Brown

e

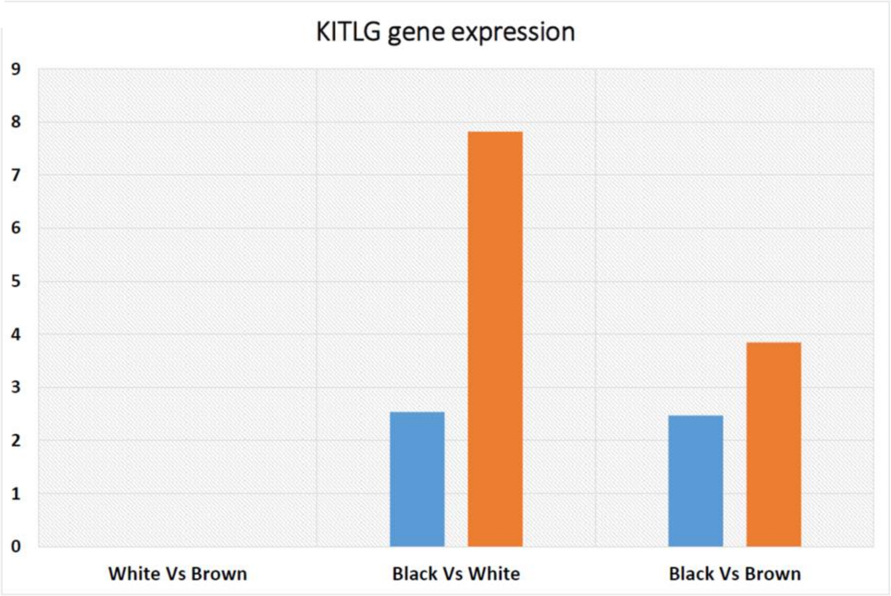
KITLG gene expression
9
8
7
6
5
4
3
2
1
0
White Vs Brown
Black Vs White
Black Vs Brown

f

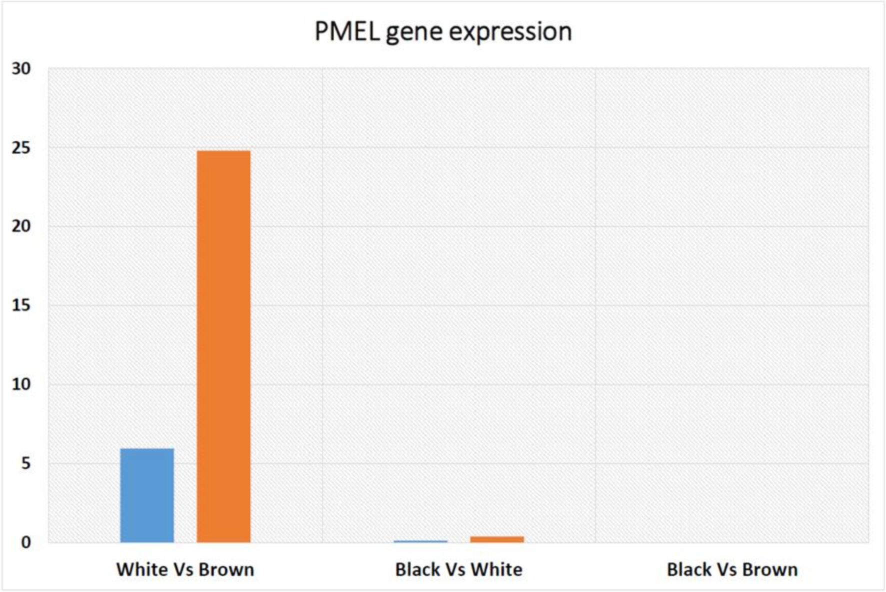
PMEL gene expression
30
25
20
15
10
5
0
White Vs Brown
Black Vs White
Black Vs Brown

g

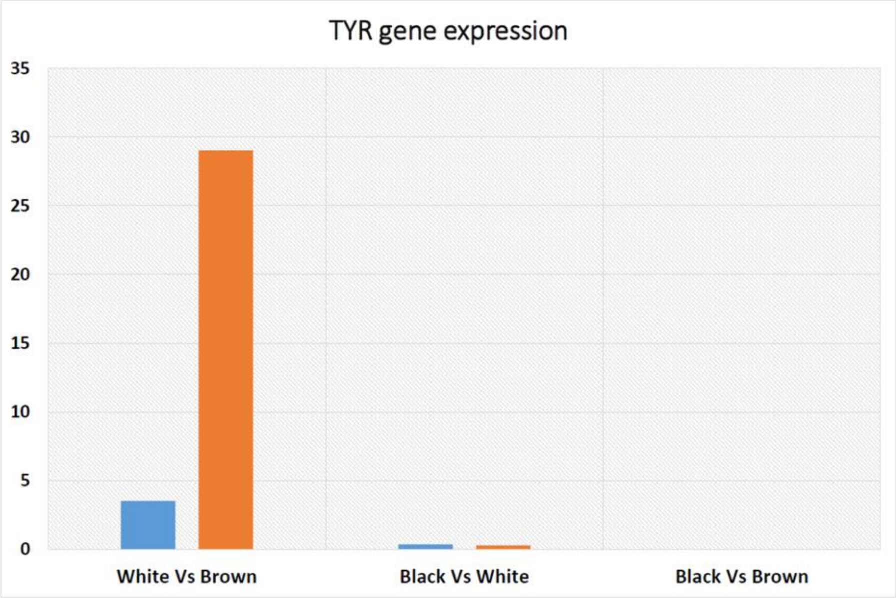

TYR gene expression
35
30
25
20
15
10
5
0
White Vs Brown
Black Vs White
Black Vs Brown

h

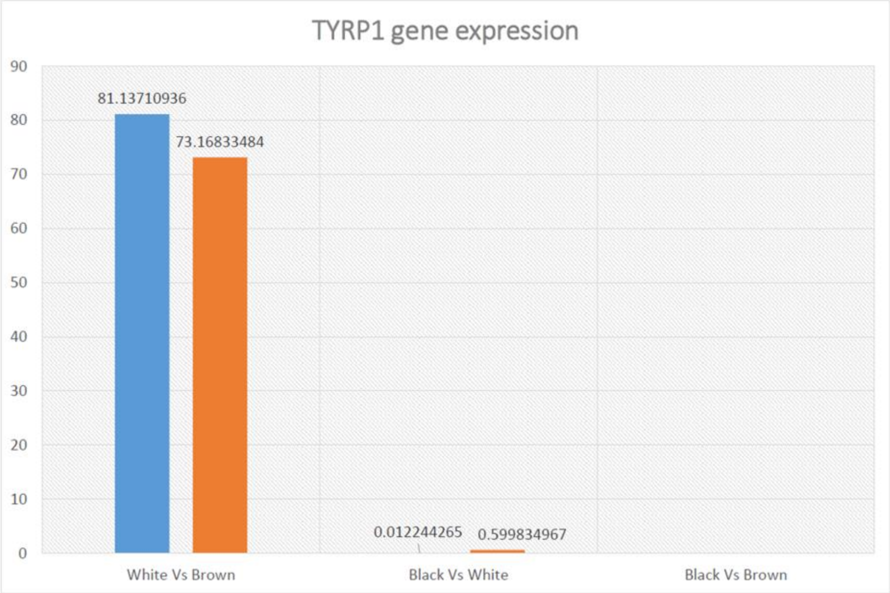

TYRP1 gene expression
90
80
70
60
50
40
30
20
10
0
81.13710936
73.16833484
0.012244265
0.599834967
White Vs Brown
Black Vs White
Black Vs Brown

i

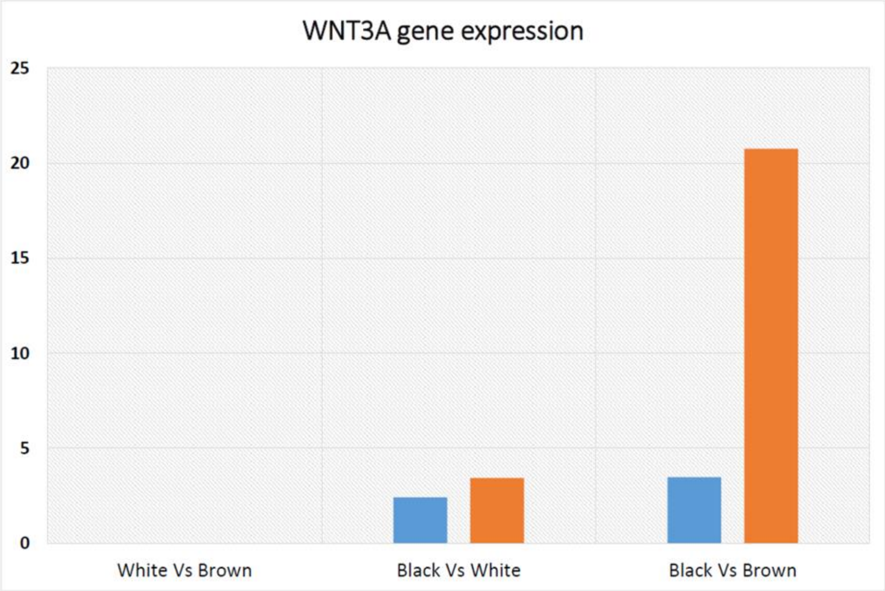

WNT3A gene expression
25
20
15
10
5
0
White Vs Brown
Black Vs White
Black Vs Brown

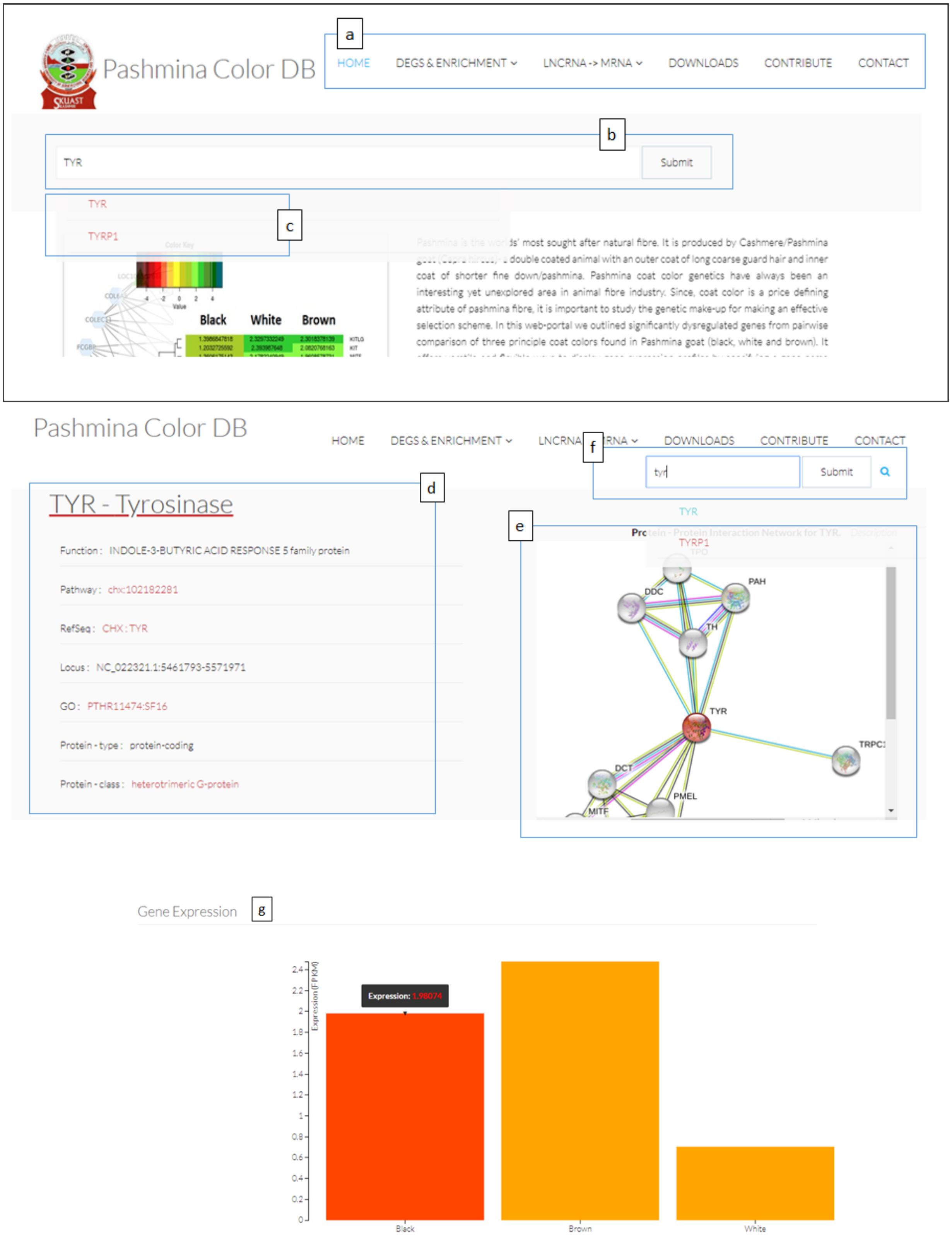

Fig. 2: Web interface for Pashmina color database.

(a) Navigation panel: There are six tabs provided at the top of the web interface (*'Home', 'DEGs & Enrichment', 'lncRNA –> mRNA', 'Downloads', 'Contribute'* and *'Contact'*) through which users can navigate and explore the required information. '*DEGs & Enrichment'* tab contain *i)* differentially expressed genes between different groups, *ii)* Combined and group specific enrichment data, *iii)*

relation between different pathways and GO terms. *lncRNA –> mRNA* contain information about lncRNAs and their target genes in *cis* and *trans* configuration.

(b) Search panel available at homepage: Text query box is provided at the top of each page to search for expression data using Gene name.

(c) Ajax support for quick data retrieval and browse through all available genes matching input query.

(d) Gene information tab: Contain general information about gene (annotation, protein class, pathways etc.,),

(e) Protein-protein interaction map

(f) Search panel at the top of each page

(g) Gene expression in different samples.

Table 7: Potential target genes of lncRNAs in *trans* – configuration that are involved in melanogenesis.

Protein Coding gene	lncRNAs in trans - configuration
**ASIP**	*-LOC102180344, LOC102181394, -LOC102186545, LOC106502376, LOC106503620,*
**FZD4**	*LOC102179411, LOC102180344, LOC102181394, -LOC102186545, LOC106502376*
**WNT3A**	*-LOC102186545, -LOC106503620*
**KIT**	-LOC102179411, *LOC102180344, -LOC102181394, -LOC102186545, LOC106502376*
**TYR**	*-LOC102169815, -LOC102172600, -LOC102177771, -LOC102184574, -LOC106502238, LOC106502886, -LOC106503063, -LOC106503218, -LOC106503370*
**TYRP1**	-LOC102169221, *-LOC102169815, -LOC102172600, -LOC102177771, -LOC102184574*, LOC102184958, *-LOC106502238,* LOC106502859, LOC106503694, *-LOC106503370,* LOC106502886, *-LOC106503063, -LOC106503218*
**DCT**	*-LOC102169815, -LOC102172600*
**PMEL**	-LOC102169221, *-LOC102177771, -LOC102184574,* LOC102184958, *-LOC106502238,* -LOC106502859, *LOC106502886, -LOC106503063, -LOC106503218, -LOC106503370,* LOC106503694

Negative sign implies negative correlation between target mRNA and lncRNA. Italic font indicates that one lncRNA acts on at least two different mRNAs involved in melanogenesis.
